# The role of YAP/TAZ activity in cancer metabolic reprogramming

**DOI:** 10.1186/s12943-018-0882-1

**Published:** 2018-09-03

**Authors:** Xiaodong Zhang, Haiying Zhao, Yan Li, Di Xia, Liang Yang, Yingbo Ma, Hangyu Li

**Affiliations:** 1 Department of General Surgery, The Fourth Affiliated Hospital of China Medical University, 4 Chongshan East Street, Shenyang, 110032 China; 2 Department of Gynecology, Beijing Obstetrics and Gynecology Hospital, Capital Medical University, Beijing, China

**Keywords:** YAP/TAZ, Metabolic reprograming, Glycolysis, Gluconeogenesis, Fatty acids, Mevalonate, Glutamine

## Abstract

In contrast to normal cells, which use the aerobic oxidation of glucose as their main energy production method, cancer cells prefer to use anaerobic glycolysis to maintain their growth and survival, even under normoxic conditions. Such tumor cell metabolic reprogramming is regulated by factors such as hypoxia and the tumor microenvironment. In addition, dysregulation of certain signaling pathways also contributes to cancer metabolic reprogramming. Among them, the Hippo signaling pathway is a highly conserved tumor suppressor pathway. The core oncosuppressive kinase cascade of Hippo pathway inhibits the nuclear transcriptional co-activators YAP and TAZ, which are the downstream effectors of Hippo pathway and oncogenic factors in many solid cancers. YAP/TAZ function as key nodes of multiple signaling pathways and play multiple regulatory roles in cancer cells. However, their roles in cancer metabolic reprograming are less clear. In the present review, we examine progress in research into the regulatory mechanisms of YAP/TAZ on glucose metabolism, fatty acid metabolism, mevalonate metabolism, and glutamine metabolism in cancer cells. Determining the roles of YAP/TAZ in tumor energy metabolism, particularly in relation to the tumor microenvironment, will provide new strategies and targets for the selective therapy of metabolism-related cancers.

## Background

Metabolism is a basic characteristic of cellular activities, including material metabolism and energy metabolism. Aerobic oxidation of glucose is the main energy supply for normal cells, whereas tumor cells prefer anaerobic glycolysis to maintain their growth and survival, even in the presence of sufficient oxygen, which is known as the “Warburg effect” [1]. Cancer metabolic reprogramming not only provides ATP for tumor cells, but also provides essential macromolecules for its protein and nucleotide biosynthesis. In recent years, studies have found that tumor cell metabolic reprogramming is regulated by many different factors [2, 3], such as hypoxia and the tumor microenvironment. Under hypoxia or an inflamed microenvironment, the tumor cells significantly increase their glucose uptake and lactate production levels. In addition, the expression levels of key enzymes in the metabolic process are upregulated, such as pyruvate kinase 2 (PKM2) and glucose transporter (GLUTs). In addition, dysregulation of certain signaling pathways also contributes to cancer metabolic reprogramming [4–6]. However, the details of the regulatory mechanism of metabolic reprogramming in tumor cells remain unclear.

**Fig. 1:**
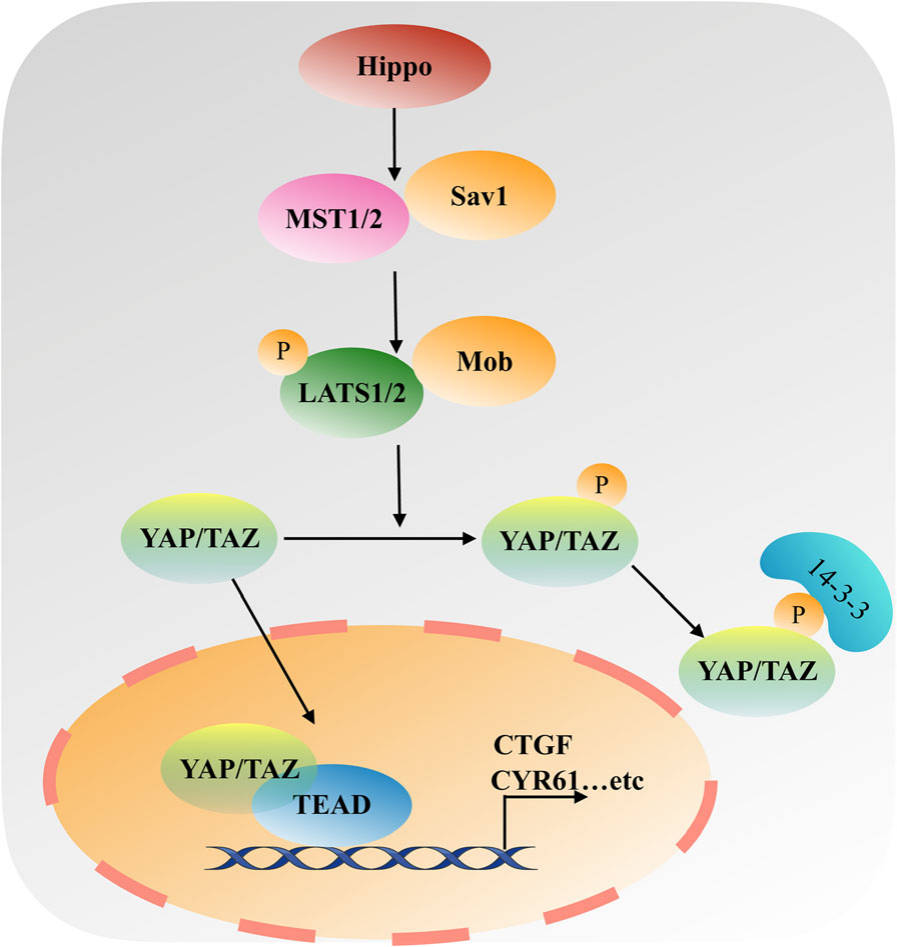
A simplified illustration of HIPPO signaling pathway. The Hippo signaling pathway is mainly comprised of MST1/2, Sav1, LATS1/2, Mob, YAP and/or its paralog TAZ. When the Hippo pathway is “ON”, MST1/2 phosphorylates and activates LATS1/2, which in turn phosphorylates YAP/TAZ and inhibits YAP/TAZ activity, leading to YAP/TAZ cytoplasmic retention and binding to 14–3-3 proteins or proteasomal degradation. When the Hippo signaling pathway is “OFF”, MSAT1/2 and LATS1/2 are inactivated, the transcriptional coactivators YAP/TAZ cannot be phosphorylated by LATS1/2 and freely translocate to nucleus and bind to TEAD transcription factors, promoting the expression of downstream target genes, such as CTGF and CYR61, which are involved in growth, proliferation, and survival

The Hippo signaling pathway is a highly conserved tumor suppressor pathway, which mainly comprises mammalian Ste20-like kinases 1/2 (MST1/2) and large tumor suppressor 1/2 (LATS1/2), yes association protein (YAP) and/or its paralog TAZ (also known as WW domain containing transcription regulator 1 (WWTR1). MST1/2 and LATS1/2 are two oncosuppressive kinases. When the Hippo pathway is activated, MST1/2 phosphorylates and activates LATS1/2, which in turn phosphorylates YAP/TAZ and inhibits YAP/TAZ activity (Fig. 1). Inactivation of this pathway is closely related to the occurrence and development of multiple tumors [7]. Most studies have found that YAP/TAZ are abnormally overexpressed in tumors and promote tumorigenesis, and considered as carcinogenic genes in many solid cancers [8–10], even though some evidences indicate that YAP/TAZ functions as tumor suppressors [11, 12]. Immunohistochemical staining revealed that the high expression of YAP/TAZ was mainly detected in the tumor cell nuclei [13–16]. So it indicates that the oncogenic role of YAP/TAZ mainly depends on their activity and nuclear localization. The tyrosine or serine phosphorylation of YAP/TAZ or the lysine monomethylation of YAP/TAZ may contribute to YAP/TAZ cytoplasmic retention [17–19]. In nucleus, the transcriptional coactivators YAP/TAZ mainly depend on multiple domains to interact with TEA domain (TEAD) transcription factors because of the lack of DNA-binding domains in YAP/TAZ [20, 21]. YAP/TAZ and TEAD form a complex in the nucleus to promote the expression and activation of downstream target genes. Recent studies have shown that Hippo pathway does not exclusively regulate YAP/TAZ phosphorylation and nuclear translation. Instead, other signaling pathways also induce YAP/TAZ activation and nuclear localization at transcriptional and post-translational levels, such as Wnt/β-catenin signaling pathway [22, 23], JNK signaling pathway [24] and Rho-GTPs signaling pathways [25], even in tumor cell energy metabolism [26, 27]. Thus, YAP/TAZ function as key nodes of multiple signaling pathways and serve as nuclear and transcriptional mediator to directly mediate target genes transcription in most cancer cells.

The present review examines the regulatory mechanism of YAP/TAZ on glucose metabolism, fatty acid metabolism, mevalonate metabolism, and glutamine metabolism in cancer cells, and provides new concepts in our understanding of cancer metabolic reprogramming and its related molecular mechanisms.

## Main text

### The role of YAP/TAZ in glucose metabolism

#### YAP/TAZ in anaerobic glycolysis

**Fig. 2:**
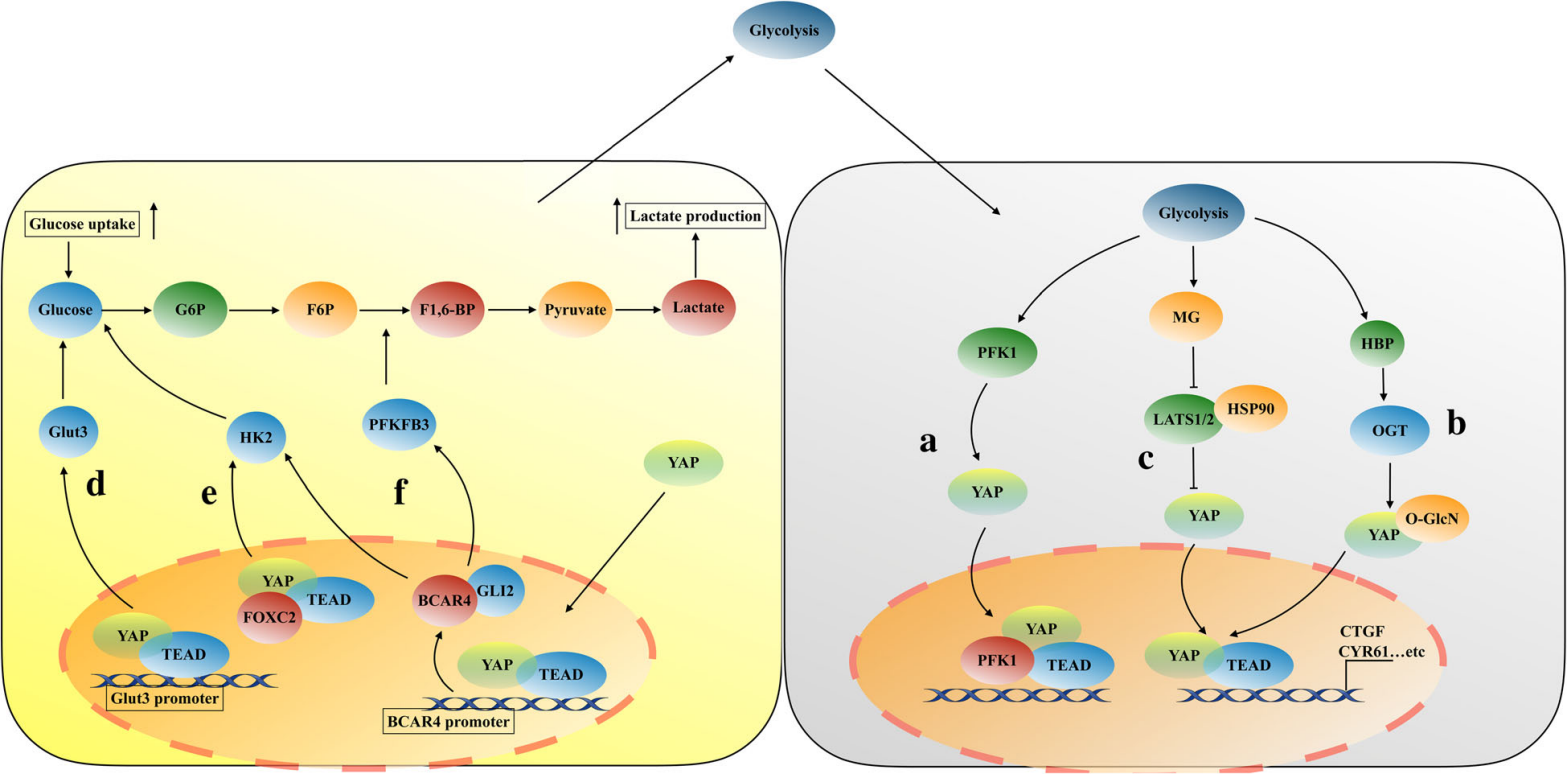
A simplified illustration of YAP/TAZ and glycolysis. (a). Glycolysis upregulates the activity of PFK1 (phosphofructokinase) to promote YAP/TAZ transcriptional cooperation with TEAD factors, and form a PFK1-TEAD1-YAP/TAZ complex in cells nucleus. (b). Glycolysis activates YAP through the HBP (hexosamine biosynthesis pathway). YAP is O-GlcNAcylated by OGT (O-linked b-N-acetylglucosamine transferase). O-GlcNAcylation of YAP promotes its nuclear translocation and transcriptional activity. (c). MG (Methylglyoxal), a side-product of glycolysis, promotes YAP transcriptional cooperation with TEAD factors by reducing the binding of HSP90 and LATS1 and inhibiting LATS1 activity. (d). YAP-TEAD binds with the GLUT3 promoter to directly regulate the transcription of GLUT3 and then promotes glycolysis in tumor cells. (e). FOXC2 (forkhead box protein C2) interacts with YAP and TEAD in cells nucleus to activate YAP, and then the activation of YAP upregulates the expression of HK2 to promote cells glycolysis. (f) YAP-TEAD directly binds with the two site (GGAATT/GGAATC) in the promoter region of lncRNA BCAR4 to upregulate the expression and transcriptional activity of HK2 and PFKFB3 to promote cells glycolysis

Unlike normal cells, tumor cells rely mainly on glycolysis to provide energy and substances necessary for sustained cell proliferation, even under normoxic conditions, which is called Warburg effect [28]. Enzo et al. found that the transcriptional activity of YAP/TAZ is regulated by glucose metabolism and does not rely on the hexosamine biosynthetic pathway or protein glycosylation [29]. When YAP/TAZ are fully active, the cells increase their glucose uptake and rate of glycolysis. While inhibition of glucose metabolism or a reduction in glycolysis induces a decrease in YAP/TAZ transcriptional activity [29]. Further studies showed that the key enzyme of glycolysis, phosphofructokinase 1 (PFK1), plays an important role in this regulation (Fig. 2a) [29]. Knockout of *PFK1* significantly inhibits YAP/TAZ activity. Mechanistically, in the presence of glycolysis, PFK1 binds the transcription factor TEAD1 to stabilize the binding of YAP/TAZ and TEAD1. Subsequently, PFK1-TEAD1-YAP/TAZ forms a complex in the nucleus, which is observed to promote the malignant biological behavior of breast cancer cells. This finding indicates that YAP/TAZ’s oncogenic activity could be unleashed by anaerobic glycolysis in some cancer cells undergoing metabolic reprogramming. However, two recent reports have revealed a novel post-transcriptional modification of YAP regulated by the hexosamine biosynthesis pathway (HBP) in response to metabolic nutrients (Fig. 2b) [30, 31]. The HBP is an important glucose metabolism pathway, which controls metabolic flux and O-GlcNAcylation. In high glucose conditions, O-GlcNAc transferase (OGT), which is a key enzyme of the HBP, O-GlcNAcylates YAP at different O-GlcNAc sites, such as Ser109 and Thr241, while the TAZ could not be O-GlcNAcylated. YAP O-GlcNAcylation promotes its expression, enhances its stability, prevents its phosphorylation, and activates its transcriptional activity [30, 31]. Mechanistically, Peng et al. found that YAP O-GlcNAcylation prevents LATS1-induced YAP phosphorylation by directly blocking its interaction with LATS1, the O-GlcNAcylation of YAP does not compete with phosphorylation at serine 109, it indicates that perhaps glycosylation is the main modification and functional regulator rather than phosphorylation at serine 109 [30]. In contrast, Zhang et al. revealed that O-GlcNAcylation of YAP at Thr241 antagonizes LATS1-mediated phosphorylation of YAP at Ser127, which promotes YAP transcriptional activity; Moreover, YAP is O-GlcNAcylated on its second WW domain, while TAZ has only one WW domain that might not be O-GlcNAcylated, and this may support why YAP is more important than TAZ [31]. Interestingly, both of the two reports have uncovered a positive feedback loop between YAP and cellular O-GlcNAcylation. The novel modification of YAP O-GlcNAcylation will be a potential therapeutic intervention target for cancer associated with high blood glucose levels.

Methylglyoxal (MG), a side-product of glycolysis, could also activate YAP and promote the growth and metastasis in breast cancer cells (Fig. 2c) [32]. In breast cancer tissues, high level of MG is positively correlated with high expression of YAP, which is localized in cell nucleus. In addition, elevated endogenous MG levels contribute to YAP localization in the nucleus and increase YAP transcriptional activity in breast cancer cells [32]. The activation of YAP is mainly dependent on the inhibition of LATS1 kinase. Of note, LATS1 kinase is also a client of Hsp90 chaperone protein, and its expression level and activity are dependent on Hsp90 [33]. Inhibition of Hsp90 decreases LATS1 kinase stability and promotes LATS1 kinase degradation [33]. Therefore, a further mechanistic study found that MG induces post-translational glycation of Hsp90 and inactivated Hsp90, which in turn affects LATS1/2 protein stability and induces LATS1/2 kinase degradation, decreases expression of LATS1/2 then promotes YAP nuclear translocation and oncogenic activity [32]. Anaerobic glycolysis is usually considered as a downstream consequence of tumor development and can be induced by oncogenes; however, these findings suggest that glycolysis could promote tumor malignancy by regulating certain oncogenic signals, such as those induced by YAP/TAZ.

In addition to the regulation of YAP/TAZ activity by glycolysis, YAP/TAZ activation also promotes glycolysis in tumor cells. YAP-5SA (a mutant that lacks S61, S109, S127, S164, and S381 five reported LATS phosphorylation sites) is used to stably activate YAP [34], which led to a significant increase in glucose uptake and lactate production in the cell culture medium, and the YAP-5SA cell medium also shows a lower pH value [35]. Besides, active YAP promotes the transcription activity and expression of the key glycolysis enzyme GLUT3, probably via the conserved TEAD-binding site in the *GLUT3* promoter (Fig. 2d). This finding indicates that YAP could promote glycolysis in tumor cells by directly regulating the transcription of *GLUT3* [35]. As shown in Fig. 2e, Song et al. found that YAP is positively regulated by forkhead box protein C2 (FOXC2). Activation of YAP specifically elevates the expression of Hexokinase 2 (HK2) at both mRNA and protein level [36]. Functionally, FOXC2 acts as a bridge to interact with YAP and TEAD, and forms a FOXC2-YAP-TEAD complex, which leading to activation of HK2 and eventually promote glycolysis in nasopharyngeal carcinoma cells [36]. However, the FOXC2-YAP-TEAD complex does not bind to the promoter region of HK2, even though this complex has a positive effect on the HK2 transcriptional regulation. This mechanism remains controversial. Gao and colleges found that YAP/TEAD/p65 complex binds to the promoter region of HK2 to synergistically regulate HK2 transcription and ultimately promotes glycolysis in breast cancer cells [37]. Bringing together the two observations, it will be important to understand under different conditions, YAP/TEAD may co-operate with different transcriptional factors to regulate the target gene expression in different way. Another mechanism for the regulation of glycolysis by YAP is demonstrated recently. In breast cancer cells, long non-coding RNA breast cancer anti-estrogen resistance 4 (BCAR4) coordinates with the GLI2-dependent Hedgehog signaling to mediate YAP-induced glycolysis, and forms a YAP-BCAR4/GLI2-glycolysis axis [38]. Mechanistically, BCAR4 is a direct transcriptional target gene of YAP (Fig. 2f). The promoter region of BCAR4 has two YAP binding sites (GGAATT/GGAATC). YAP promotes BCAR4 transcription by directly binding with the two sites in the promoter region of BCAR4. Subsequently, BCAR4 activates Hedgehog effector GLI2 and forms a BCAR4/GLI2/p300 complex, which directly activates the transcription of downstream target glycolysis-related genes HK2 and PFKFB3 (6-phosphofructo-2-kinase/fructose-2, 6-biphosphatase 3) through acetylation of H3K27ac histones, and ultimately promotes the glycolysis of breast cancer cells [38]. The identification of lncRNAs mediated regulation of glycolysis by YAP has improved our understanding of the regulatory mechanism between YAP and glycolysis, providing a new target for the targeted therapy of glycolysis-related diseases.

Collectively, these findings indicated that there might be a positive feedback loop between glycolysis and YAP/TAZ. On one hand, glycolysis activates YAP/TAZ transcriptional activity by promoting the expression of key glycolysis enzymes. On the other hand, YAP/TAZ exerts its oncogenic functions to increase glycolysis. It seems here that YAP/TAZ is a key metabolic hub in the regulation of glycolysis. And YAP/TAZ may also represent an ideal therapy target for cancer. However, the precise mechanism of YAP/TAZ’s involvement in glycolysis is still at an early stage, and many further outstanding questions need to be answered.

#### YAP/TAZ in gluconeogenesis

Gluconeogenesis is another important component of glucose metabolism, and gluconeogenesis disorder is closely related to the development of malignant tumors and insulin resistance diseases, such as diabetes and nonalcoholic fatty liver disease [39]. Gluconeogenesis is regulated by insulin and glucagon, with the involvement of many transcription factors, such as factor FoxO1 (forkhead transcription factor 1, FoxO1) [40], CREB (cAMP-response element binding protein, CREB) [41], PGC-1 (peroxisome proliferator activated receptory coactivator-1, PGC-1) [42], G-6-Pase (glucose-6-phosphatase, G-6-Pase) and PEPCK (phosphoenolpyruvate carboxykinase, PEPCK) [43, 44]. Glucagon positively regulates gluconeogenesis and stimulates net hepatic gluconeogenic flux via activation protein kinase A (PKA) in a cAMP-dependent manner, which in turn activates a variety of transcription factors, such as PGC-1 alpha and ultimately promotes the expression of gluconeogenesis genes [45].

Recently, it is observed that a high level of glucagon inhibits the expression and activity of YAP/TAZ [46, 47]. Glucagon increases cAMP levels by binding G-protein coupled receptor (GPCR). Accumulation of cAMP activates protein kinase A (PKA), which in turn inhibits Rho GTPase. This subsequently activates LATS1/2, which is a key upstream regulatory factor of YAP/TAZ [46, 47]. Activation of LATS1/2 further phosphorylates YAP at Serine 127, resulting in YAP retention in the cytoplasm and loss of transcriptional activity, ultimately leading to its degradation [46, 47]. Yue et al. showed that YAP suppresses gluconeogenesis in a PGC1α-dependent manner in primary hepatocytes [48]. Mechanistically, YAP mainly suppresses the expression of gluconeogenic genes *PCK1* and *G6PC* in response to glucagon by inhibiting the ability of PGC1α binding to the promoters of *PCK1* and *G6PC* [48]. Interestingly, activation of YAP only reduces the expression levels of *PGC1α* mRNA, but did not affect the PGC1α protein level [48]. However, the exactly mechanism on how YAP regulates PGC1α is still unclear. Yue and colleagues thought that YAP may inhibit PGC1α in an indirect way and S6 kinase may be one potential indirect mediator [48]. Therefore, further more studies are needed to demonstrate the exactly effect of YAP on PGC1α, and it will provide a new molecular mechanism for YAP to regulate glucose metabolism.

### The role of YAP/TAZ in lipid metabolism

Fatty acids de novo synthesis is one of the most important metabolic hallmarks in cancer cells. Enhanced lipogenesis provides an important source of material and energy for the growth of tumors [49]. This lipogenic conversion induces high expression and activity of key enzymes involved in the fatty acid synthesis in tumor cell, such as acetyl coenzyme A carboxylase (acetyl-CoA carboxylation, ACC) and fatty acid synthase (FAS) [50–53], which in turn promotes cancer cell proliferation and survival.

**Fig. 3:**
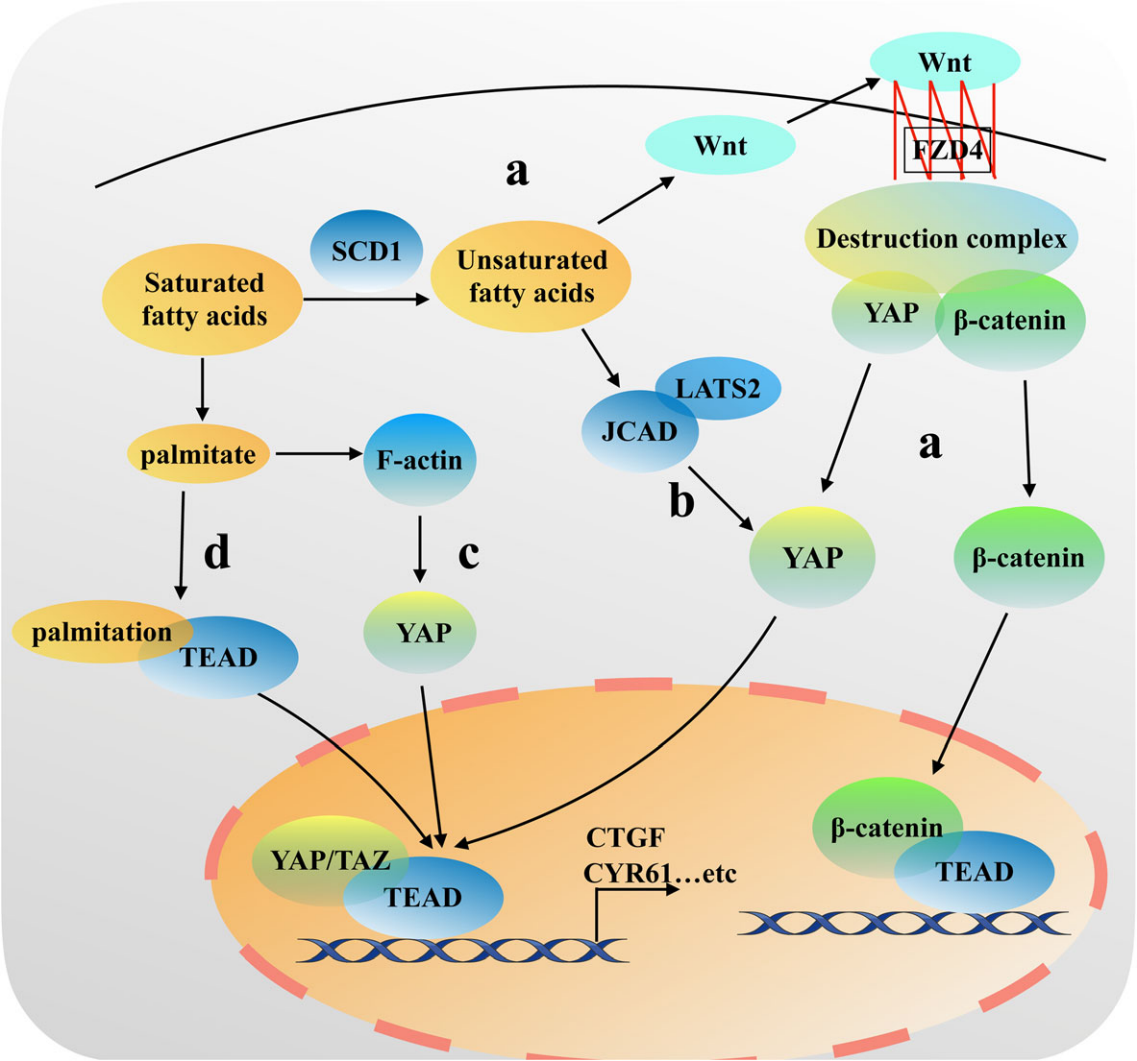
A simplified illustration of YAP/TAZ and fatty acids. (a). SCD1 promotes the synthesis of unsaturated fatty acids. Unsaturated fatty acids activate Wnt ligand. Activation of the Wnt ligand combined with FZD4 receptor to damage the destruction complex, ultimately stabilize β-catenin and YAP/TAZ protein activity and promote β-catenin and YAP/TAZ accumulation in the nucleus to play the function role of transcription regulation. (Destruction complex: APC, Axin1, GSK3, β-TrcP). (b). Free fatty acid induces high expression of JCAD, which in turn binds to the domain of LATS2 kinase and inhibits the ability of LATS2 to phosphorylate YAP, leading to activate YAP transcription by dephosphorylating and promote YAP nuclear translocation to promote hepatoma cell proliferation. (c). Palmitate promotes YAP transcriptional activity in a F-actin-dependent manner. (d). Palmitate attaches to TEAD cysteine residues to palmitoylate TEAD, stabilizes TEAD binding to YAP/TAZ and promotes their transcriptional activity

Stearoyl coenzyme A desaturase 1 (SCD1) is a key enzyme involved in mono-unsaturated fatty acids synthesis, which shifts saturated fat acid synthesis to unsaturated fatty acid synthesis. Several evidences suggest that SCD1 is positively relation with a variety of malignant tumors [54–56]. A recent study reveals that SCD 1 promotes nuclear localization and transcriptional activity of YAP/TAZ to regulate lung cancer stemness (Fig. 3a) [57]. The regulation of YAP/TAZ by SCD1 is at least in part dependent on Wnt/β-catenin pathway activity, but not dependent on the Hippo signaling pathway. Confirming prior reports, YAP and TAZ are integral components of the β-catenin destruction complex [58]. When Wnt is on, YAP/TAZ are released from the complex, translocate to nuclear and exert their functions in transcription regulation (Fig. 3a) [58]. Noto et al. documented that SCD1 mediates the release of β-catenin and YAP/TAZ from the β-catenin destruction complex via activation Wnt ligands, which induced by increasing the synthesis of large amounts of unsaturated fatty acids (Fig. 2). This in turn promotes β-catenin and YAP/TAZ accumulation in the nucleus to promote their target genes transcription (Fig. 3a) [57].

Besides the enzymes in the fatty acid, free fatty acids can also regulate YAP/TAZ transcriptional activity. In nonalcoholic fatty liver disease-associated hepatocellular carcinoma (NASH-HCC), the overload of free fatty acid induces a significantly high expression of junctional protein-associated with coronary artery disease (JCAD) [59]. JCAD overexpression activates YAP transcription by dephosphorylating and promoting YAP nuclear translocation (Fig. 3b). In this scenario, JCAD binds to the domain of LATS2 kinase and inhibits the ability of LATS2 to phosphorylate YAP, which in turn activating YAP to promote hepatoma cell proliferation (Fig. 3b) [59]. In concert, palmitate, a saturated free fatty acid, also promotes YAP transcriptional activity in a F-actin-dependent manner in β-cells (Fig. 3c) [60]. In addition, a recent novel and important study firstly reveals that palmitate is involved in a post-translational modification, which is called protein S-palmitoylation, via attachment to TEAD cysteine residues to indirectly regulate the transcription of YAP/TAZ (Fig. 3d) [61]. Chan et al. found that TEAD family transcription factors are autopalmitoylated at C359 in a PATs-independent manner, which is required for binding to YAP/TAZ. Palmitoylation of TEAD stabilizes its binding to YAP/TAZ and promotes their transcriptional activity (Fig. 3d) [61]. This may provide a potent treatment strategy for cancer by disrupting TEAD-YAP interaction.

Together, these observations suggest that lipid metabolism might regulate YAP/TAZ activity, yet the researches are limited and the precise mechanism remains unclear. Moreover, it would also be interesting to investigate whether YAP/TAZ could regulate lipid metabolism in cancer cells, such as lipogenesis and lipolysis.

### The role of YAP/TAZ in mevalonate metabolism

The mevalonate metabolic pathway is an important pathway that mainly uses acetyl coenzyme A as the raw material to synthesize sterols and other nonsteroidal lipids. Some intermediates of mevalonate metabolism, such as farnesyl pyrophosphate (FPP) and geranylgeranyl pyrophosphate (GGPP), are directly involved in protein prenylation, which is vital to maintain cell and lipid metabolism activities. Indeed, disruption of the mevalonate metabolic pathway leads to the occurrence of multiple tumors [62–64].

**Fig. 4:**
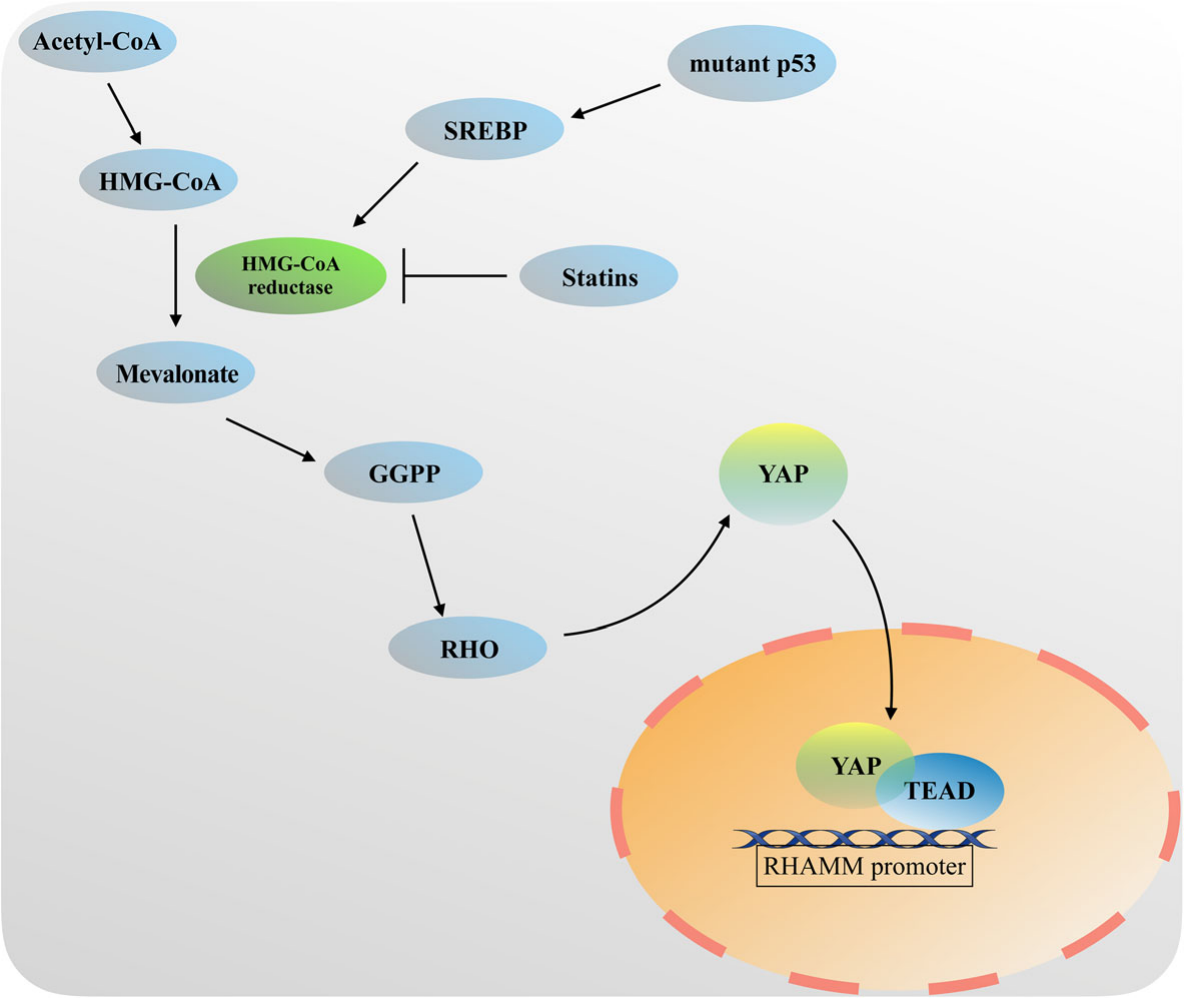
A simplified illustration of YAP/TAZ and mevalonate. HMG CoA produces mevalonate through the activity of the HMG CoA reductase (HMGCR). Geranylgeranyl pyrophosphate (GGPP), the intermediate of mevalonate metabolism, activates RHO to promote YAP/TAZ transcriptional cooperation with TEAD factors in cells nucleus. Then YAP/TAZ-TEAD binds the specific sites in the RHAMM promoter to play the function role of transcription regulation. The transcription of HMG CoA reductase (HMGCR) also can be inhibited by Statins or activated by SREBP transcription factors, which can be upregualted by mutant p53. This explains why some small-molecule inhibitors such as statins could inhibit the nuclear localization and transcriptional activity of YAP/TAZ

YAP/TAZ is a downstream transcriptional coactivating factor in the Hippo signaling pathway, and its activity is also regulated by mevalonate metabolic pathway. As shown in Fig. 3, when small-molecule inhibitors, such as statins, are used to inhibit the activity of HMG-COA reductase in the mevalonate pathway, the nuclear localization and transcriptional activity of YAP/TAZ are also inhibited [65]. In addition, GGPP plays an important role in this process. GGPP promotes YAP/TAZ nuclear localization and increases YAP/TAZ transcriptional activity via activation of Rho GTPases (Fig. 4) [65]. And activation of Rho GTPases regulated by Mevalonate also promotes YAP/TEAD to bind to hyaluronan-mediated motility receptor (*HMMR*; also known as RHAMM) promoter at two specific TEAD-binding sites, which in turn activates RHAMM transcription [66]. Interestingly, the regulation of YAP/TAZ by Rho GTPases is largely independent of the LATS1/2 Hippo pathway kinases, instead relying on YAP/TAZ phosphorylation [65, 66], However, the conclusion is in conflict with the previous reports that RHO induces YAP activity in a LATS1/2-depandent manner [46, 47]. Bringing together those observations, one of the reasons may be that Rho GTPases regulate YAP/TAZ activity via inhibition an unknown kinase in mevalonate metabolic pathway. However, it remains a issue to identity the relevant kinase. Another reason may be that Rho GTPases-induced YAP/TAZ activation in mevalonate pathway is distinct from YAP/TAZ activation by the cytoskeleton accumulation through inhibition of LATS kinase activity, while it is controversial whether Rho GTPases affect F-actin polymerization to regulate YAP/TAZ activity in mevalonate metabolic pathway. In addition, in a breast cancer cell line, YAP/TAZ could also be activated by SREBPs (sterol regulatory element-binding proteins), which is the main method of regulation of the mevalonate pathway [65]. Moreover, mutant p53 also promotes YAP/TAZ activity and contributes to cancer cell malignancy by sustaining SREBP expression in the mevalonate metabolic pathway (Fig. 3) [65].

These in vivo and in vitro studies confirmed a new mechanism of mevalonate regulation of YAP/TAZ expression and transcriptional activity, revealing a possible process in which statins play an anticancer effect and also providing some potential targets to develop cancer treatment drugs.

### The role of YAP/TAZ in glutaminolysis

The tricarboxylic acid cycle (TCA cycle) acts as the central metabolic hub and provides a majority of the energy and necessary biosynthetic precursors used by cell [67]. In order to maintain a functional TCA cycle, cancer cells often rely on elevated glutaminolysis. Therefore, glutaminolysis is another important characteristic of the energy metabolism in tumors [68]. Glutaminolysis catabolizes glutamine to yield glutamate and ammonia through the initial deamination of glutamine by glutaminase (GLS). Then glutamate is converted to α-KG, a TCA cycle intermediate, by either glutamate dehydrogenase (GDH) or transaminases [69, 70]. The major function of glutaminolysis is not only to supply α-KG to replenish the TCA cycle and generate ATP, but also provides nitrogen and anabolic carbons required for the synthesis of proteins, nucleotides and lipids macromolecule for the growth and proliferation of cancer cells [69, 70].

**Fig. 5:**
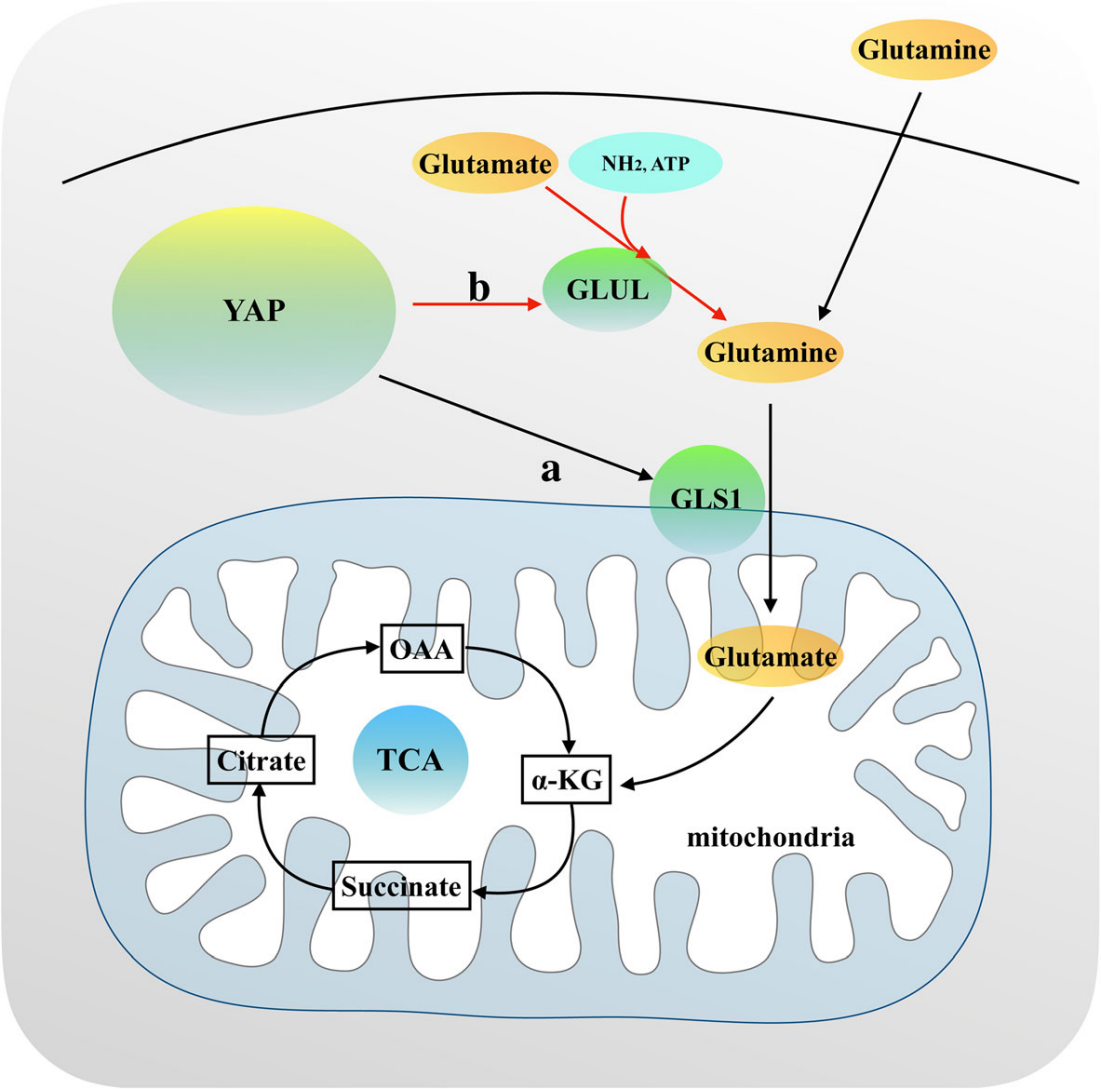
A simplified illustration of YAP/TAZ and glutaminolysis. (a). Activation of YAP/TAZ upregulates the expression of glutamine by promoting the expression and transcriptional activity of glutamine synthetase (GLUL). (b). YAP/TAZ upregulates the expression of glutaminase (GLS1) to promote glutaminolysis

Recently, a connection between glutaminolysis and YAP/TAZ activity is observed. Activation of YAP/TAZ upregulates the level of glutamine, enhances the de novo nucleotides synthesis pathway, and induces the liver size to increase by promoting the expression and transcriptional activity of glutamine synthetase (GLUL) (Fig. 5a) [71]. As shown in Fig. 5b, Bertero et al. found that YAP/TAZ plays an important role in the regulation of glutamine metabolism and glycolysis in pulmonary arterial hypertension [72]. Vascular stiffness activates YAP/TAZ-dependent glutaminolysis mechanically to drive pulmonary hypertension. This activation of YAP/TAZ modulates metabolic enzymes, including glutaminase (GLS1), to coordinate glutaminolysis and glycolysis (Fig. 5b). In endothelial cells, YAP/TAZ knockdown reduces lactate production and decreases the extracellular lactate/pyruvate ratio. YAP/TAZ knockdown also blunts the effects of a stiff extracellular matrix on intracellular glutamine, glutamate, and aspartate [72]. By contrast, stable expression of YAP increases extracellular lactate and the lactate/pyruvate ratio, decreases glutamine, and increases glutamate and aspartate. Further studies reveal that the YAP/TAZ-GLS1 axis plays an important role in YAP/TAZ-dependent glutaminolysis in pulmonary vascular endothelial cells [72]. Moreover, HIV infection-induced pulmonary hypertension is also mainly caused by the YAP/TAZ-GLS1 axis [72]. The activation of the YAP/TAZ-GLS1 axis promoted glutaminolysis and caused vascular sclerosis.

Collectively, determining the regulatory role of YAP in the metabolism of glutamine will provide the impetus for the future development of targeted therapy for pulmonary hypertension and other diseases.

## Conclusions

Glucose metabolism, fatty acid metabolism, mevalonate metabolism, and glutaminolysis not only provide energy for the growth of tumors, but also provide the necessary synthetic materials for the activities of tumor cells. Identifying and blocking the regulatory pathways and targets in the process of tumor metabolism will become a research hot spot in the development of tumor targeting therapy. In previous studies, YAP/TAZ has been identified as a signaling hub involved in the regulation of multiple signaling pathways in tumor cells, which promotes the initiation and development of many tumors [73–76]. Recent research on YAP/TAZ has demonstrated that the inhibition of the expression and transcriptional activity of YAP/TAZ could significantly inhibit the growth and invasion in tumor cells, and induces tumor cells apoptosis [77–79]. Treating tumor cells with Verteporfin, a target drugs for YAP/TAZ, significantly reversed the malignant biological behavior of the tumor cells [80–83]. Therefore, YAP/TAZ may represent ideal targets for selective tumor therapy. However, the role of YAP/TAZ in cancer metabolic reprogramming, and the specific regulatory mechanism remains unclear, thus requiring further studies. Besides, the tumor microenvironment also has a significant influence to the development of tumor cells. Hypoxia, inflammatory factors, and other microenvironment factors in the tumor microenvironment could stimulate tumor cells to undergo metabolic reprogramming. Whether YAP/TAZ have a role in in the link between tumor microenvironment and metabolic reprogramming requires further study. A deeper understanding of the role of YAP/TAZ in tumor energy metabolism will provide new strategies and targets for metabolism-related cancer therapy

## Abbreviations

FOXC2: Forkhead box protein C2
GGPP: Geranylgeranyl pyrophosphate
GLS: Glutaminase
GLUL: Glutamine synthetase
GLUT3: Glucose transporter 3
HK2: Hexokinase 2
LATS1/2: Large tumor suppressor 1/2
MG: Methylglyoxal
PFK1: Phosphofructokinase 1
PFKFB3: 6-phosphofructo-2-kinase/fructose-2, 6-biphosphatase 3
PGC-1 alpha: Peroxisome proliferator activated receptory coactivator-1
SCD1: Stearoyl coenzyme A desaturase 1
SREBPs: Sterol regulatory element-binding proteins
TCA cycle: Tricarboxylic acid cycle
TEAD: TEA domain
YAP: Yes association protein
